# Significant Remodeling Affects the Circulating Glycosaminoglycan Profile in Adult Patients with both Severe and Mild Forms of Acute Pancreatitis

**DOI:** 10.3390/jcm9051308

**Published:** 2020-05-01

**Authors:** Ewa M. Koźma, Kornelia Kuźnik-Trocha, Katarzyna Winsz-Szczotka, Grzegorz Wisowski, Paweł Olczyk, Katarzyna Komosińska-Vassev, Mariusz Kasperczyk, Krystyna Olczyk

**Affiliations:** 1 Department of Clinical Chemistry and Laboratory Diagnostisc, Faculty of Pharmaceutical Sciences, Medical University of Silesia, Katowice, Jedności 8, 41-200 Sosnowiec, Poland; kkuznik@sum.edu.pl (K.K.-T.); winsz@sum.edu.pl (K.W.-S.); vis@sum.edu.pl (G.W.); kvassev@sum.edu.pl (K.K.-V.); olczyk@sum.edu.pl (K.O.); 2 Department of Community Pharmacy, Faculty of Pharmaceutical Sciences in Sosnowiec, Medical University of Silesia, Katowice, Kasztanowa 3, 41-200 Sosnowiec, Poland; polczyk@sum.edu.pl; 3 Department of General Surgery and Multiorgan Injuries, The St. Barbara’s Specialist Hospital, Plac Medyków 1, 41-200 Sosnowiec, Poland; kasperczyk.mariusz@wp.pl

**Keywords:** acute pancreatitis, plasma glycosaminoglycans, chondroitin sulfate, heparan sulfate, hyaluronan

## Abstract

Acute pancreatitis (AP) manifests itself either as a mild, self-limiting inflammation or a severe, systemic inflammatory process that is associated with various complications and a high mortality rate. It is unknown whether these two forms of the disease can differ in the profile of circulating glycosaminoglycans, which are molecules with huge biological reactivity due to a high density of negative electric charge. Plasma glycosaminoglycans were characterized/quantified in 23 healthy controls, 32 patients with mild AP, and 15 individuals with severe disease using electrophoresis with enzymatic identification (chondroitin sulfate and heparan sulfate) or an ELISA-based test (hyaluronan). Moreover, the correlations between the glycosaminoglycan levels and clinical parameters were evaluated. Both forms of AP showed similar remodeling of the plasma profile of the sulfated glycosaminoglycans. In contrast, only in the patients with mild AP was the level of circulating hyaluronan significantly decreased as compared to the healthy controls. Both forms of AP are associated with systemic changes in the metabolism of glycosaminoglycans. However, the alterations in hyaluronan metabolism may contribute to the disease evolution. The circulating hyaluronan may have some clinical value to predict the severity of AP and to evaluate the clinical status of patients with severe AP.

## 1. Introduction

Acute pancreatitis (AP) is the most common inflammatory disease that affects the exocrine part of the pancreas. About 80% of the cases of the disease are triggered by alcohol abuse or cholelithiasis [1,2]. It is believed that at least some of the etiological factors of AP can induce an influx of calcium ions into the cytosol of the acinar cells, which leads to both a premature activation of the pancreatic enzymes in these cells and to the induction of pro-inflammatory signaling in them [3,4,5]. AP is a self-limiting mild inflammatory disease in the majority of patients. However, some patients develop a severe form of the disease, which is characterized by the occurrence of a variety of both early and late complications such as (multi)organ failure, pancreatic necrosis, pancreatic fluid collections, and/or sepsis [6,7]. Because of these complications, especially infected pancreatic necrosis and sepsis, severe AP is associated with a high mortality rate [7]. Although the detailed mechanisms that are responsible for the distinct clinical manifestation of AP remain unknown, it is believed that a key event in the pathogenesis of severe AP is the occurrence of a persistent systemic inflammatory response syndrome (SIRS), which is subsequently followed by a compensatory anti-inflammatory response syndrome [8].

**Figure 1:**
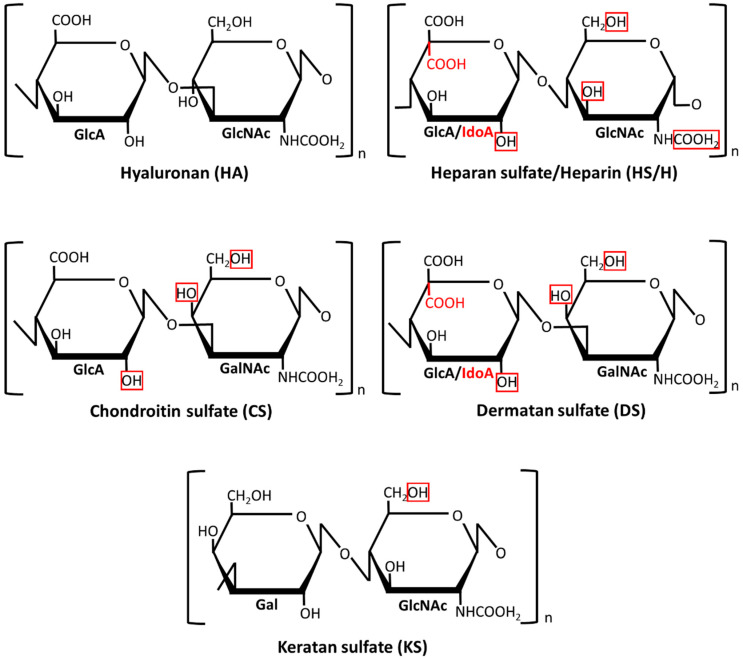
The structure of disaccharide units in the different types of glycosaminoglycans. Possible positions of sulfate groups are marked by encircling with a red line. n—polymerization degree; Gal—galactose; GalNAc—N-acetyl galactosamine; GlcA—glucoronate; GlcNAc—N-acetyl glucosamine; IdoA—iduronate.

Glycosaminoglycans (GAGs) are linear polymeric heteropolysaccharides that are widespread in all animal tissues where they are mainly located at the cell surface and in the extracellular matrix (for a review, see Reference [9]). Depending on the composition of their disaccharide monomers (Figure 1), GAGs are divided into four classes: hyaluronan (HA), heparan sulfate (HS)/heparin (H), chondroitin sulfate (CS)/dermatan sulfate (DS), and keratan sulfate (KS). In contrast to the HA that usually forms non-covalent aggregates with proteins, the remaining so-called sulfated GAGs primarily exist as chains that are covalently attached to various core proteins in the glycoproteins, which are called proteoglycans (PGs). A characteristic structural feature of GAGs is a high density of the negative electric charge that is caused by the presence of a hexuronate residue in each disaccharide unit of their chains (except for KS) and/or by the esterification of some monosaccharide residues in the disaccharide units with sulfate groups (except for HA) (Figure 1). Importantly, although both the sulfation level and the localization of the sulfate groups along the chain backbone in sulfated GAGs are most probably tissue-specific, they can change in the course of various physiological and pathological events, which is dependent on the modulating activity of the appropriate sulfotransferases. A variable sulfation pattern is a sign of the structural heterogeneity of GAGs that is also reflected in the variable size of their chains and/or a changing epimerization pattern. The last feature only concerns DS and HS/H in which the disaccharide units can contain glucuronate residue or its C5 epimer–iduronate one. The accumulation of a negative charge in the GAG chains promotes ionic interactions between them and miscellaneous proteins including those that are engaged in the initiation, progression, and modulation of inflammation such as some cell surface receptors (Toll-like receptors, cluster of differentiation 44 (CD44)), proteolytic enzymes (matrix metalloproteinases, cathepsins) and their effectors (serpins, tissue inhibitors of metalloproteinases), adhesion molecules (selectins, intercellular adhesion molecule 1), or various cytokines (for example, tumor necrosis factor (TNF)-α, interleukin (IL)-10 or many chemokines) [10,11]. Moreover, the huge biological reactivity of GAGs together with their structural heterogeneity can predestine them to be regulators/modulators in various diseases including those that are associated with inflammation. While this function of GAGs is still poorly known, several studies reported that the plasma/serum profile of these macromolecules is strongly remodeled in patients suffering from SIRS-associated diseases such as rheumatoid arthritis, systemic lupus, or sepsis [12,13,14,15,16]. These observations prompted us to examine the circulating GAGs in patients with mild and severe AP. Moreover, in order to evaluate the relevance of these molecules to AP pathogenesis, we analyzed the relationship between their plasma levels and the markers of renal and liver function and SIRS, as well as the Sequential Organ Failure Assessment (SOFA) score [17].

## 2. Experimental Section

The study protocol was approved by the local Bioethics Committee of the Medical University of Silesia in Katowice (permit no. NN-013-152/02), and written informed consent was obtained from all of the participants (both the patients with AP and healthy donors) before they were enrolled into the study. Blood samples were obtained from 47 adults who were admitted to the surgical unit of Municipal Hospital no 2 in Sosnowiec between January 2013 and September 2014 with symptoms of AP (upper abdominal pain, fever, nausea, vomiting) and serum lipase and/or amylase activity at least three-fold greater than the upper limit of the reference intervals for these enzymes [18]. All of these individuals underwent transabdominal ultrasonography at admission. Prior to their hospitalization, the patients had no symptoms of other acute or chronic inflammatory illnesses, diabetes, or chronic liver and kidney diseases. Twenty-three of our AP patients declared that their AP symptoms persisted for a maximum of three days prior to admission. The remaining individuals were admitted to hospital within the first 24 h after the symptoms appeared. Moreover, all of the included patients had AP that was caused only by the most frequent etiological factors, i.e., cholelithiasis or alcohol abuse. The organ/system function in all of these AP patients was evaluated using the SOFA score based on the measurements that were done within 24 h of admission. Organ/system failure was defined as a score of two or more for one organ or system [18]. At least one organ or system dysfunction was diagnosed in the 15 AP patients who were classified as suffering from the severe form of the disease according to the Revised Atlanta Criteria [18]. None of these patients had risk factors for a direct injury to the lungs such as pneumonia, aspiration, or lung contusion and/or symptoms of a chronic obstructive pulmonary disease at admission. However, the lung complications appeared in six of these individuals within the first 24 h after admission. Thus, these patients were classified as suffering from (moderately) severe AP, which is complicated by indirect lung injury. The AP patients who did not fulfill the Revised Atlanta Criteria for a severe form of the disease were diagnosed as patients with a mild AP. The mild and severe AP groups contained a different number of patients, which reflects the proportion between these disease forms in the population of AP patients. Blood samples were collected in ethylenediaminetetraacetic acid (EDTA)-treated vacuettes from all of the AP patients within 24 h of admission. The plasma samples that were obtained after blood centrifugation (800× *g*, 10 min, 21 °C) were stored at −75 °C until they were analyzed. The final inclusion of plasma samples to both AP groups was made after the confirmation of diagnosis based on the clinical course of the disease. The control group was composed of 23 healthy volunteers. The exclusion criteria that were applied to the control group included any symptoms of acute or chronic liver, renal and inflammatory diseases, diabetes or metabolic syndrome, cancer, or daily alcohol consumption >40 g of ethanol. Moreover, the gender and age profile of the control group was also similar to those in both AP groups. Blood samples from all of the healthy individuals were included in the analysis.

Plasma GAGs were liberated from both covalent and non-covalent complexes with proteins by exhaustive proteolysis with papain (Fluka, Buchs, Swiss) followed by β-elimination [19]. The proteolysis was conducted for 24 h at 65 °C using 25 mg of papain per 1 mL of plasma. Subsequently, 0.1 M NaOH was added to hydrolysates until pH 9 was reached, and samples were incubated for further 24 h at 40 °C. Plasma peptides that were resistant to this hydrolysis were precipitated with 7% trichloroacetic acid (Sigma-Aldrich, Steinheim, Germany) for 12 h at 4 °C and removed by centrifugation (18,000× *g*, 20 min, 4 °C). Then, the plasma GAGs were precipitated from the obtained supernatants with three volumes of cold acetone for 24 h at 4 °C and centrifuged (25,000× *g*, 20 min, 4 °C). The GAG-containing pellets were dissolved in 0.5 M CH_3_COOK, and these macromolecules were again precipitated with cold acetone and centrifuged. Subsequently, the plasma GAGs were separated from the other non-proteinic plasma components by complexation with cetylpyridinium chloride (CPC) (Loba-Chemie, Fischamend, Austria) as described previously [12]. To this purpose, the GAG solutions were incubated with 0.15% CPC for 24 h at 24 °C, and the formed CPC–GAG complexes were centrifuged (25,000× *g*, 20 min, 24 °C). Traces of CPC were removed from the GAG-containing pellets by washing with 96% ethanol.

A quantitative analysis of the GAGs that were isolated from the plasma samples was performed by measuring the hexuronate concentration according to the Blumenkrantz and Asboe-Hansen method as modified by Slim et al. [20]. Briefly, 0.1-mL aliquots of the GAG solutions were added to the samples containing 1.2 mL of 95% H_2_SO_4_ with 0.025 M sodium tetraborate, which were then placed in an ice bath. After intensive mixing the GAGs were hydrolyzed by incubating them for 7 min in a boiling bath. Subsequently, 50 µL of 0.15% *meta*-hydroxydiphenyl (Sigma-Aldrich, Steinheim, Germany) in 0.25% NaOH was added to each GAG hydrolysate. After rigorous mixing, the absorbance of samples was measured at 524 nm. The concentration of hexuronate in a particular GAG sample was calculated by interpolating the obtained absorbance values to a calibration curve that was prepared for solutions of standard d-(+) glucuronolactone (Sigma-Aldrich, Steinheim Germany). Because all of the GAG samples were measured in one day, the inter-assay variability was not estimated. In turn, the intra-assay variability of the GAG concentration was less than 7%.

In order to identify the types of hexuronate-containing GAGs, the molecules that were isolated from plasma samples were submitted to an electrophoresis on cellulose acetate before and after treatment with specific bacterial lyases. Chondroitinase ABC from *Proteus vulgaris* (Sigma-Aldrich, Steinheim, Germany) was used to degrade CS/DS. In turn, the combined action of chondroitinase ABC and heparinases I and III from *Flavobacterium heparinum* (Sigma-Aldrich, Steinheim, Germany) enabled CS/DS and HS to be eliminated, respectively. Moreover, some GAG samples were also treated with chondroitinase AC I from *Flavobacterium heparinum* (Amsbio, Abingdon, UK) in order to exclusively degrade CS. The plasma GAG treatment with chondroitinase ABC and/or heparinases was conducted in a 0.05 M Tris HCl buffer pH 7.4 containing 0.2% bovine serum albumin for 24 h at 37 °C using 0.01 IU of the former enzyme and/or 0.001 IU of heparinase I and 0.01 IU of heparinase III per 8 µg of the hexuronic acids. The GAG cleavage with chondroitinase AC I was conducted under the same buffer conditions for 2 h at 37 °C using a 0.01 enzyme unit per 8 µg of hexuronic acids. Sample components that were resistant to the enzyme degradation were precipitated with three volumes of cold 96% ethanol and subjected to an electrophoretic resolution.

Samples of the plasma GAGs (8 µg of hexuronic acids) that were untreated or degraded with the enzyme(s) were submitted to electrophoresis on Cellogel cellulose acetate strips (Serva, Heidelberg, Germany) in 0.017 M Al_2_(SO_4_)_3_. The electrophoresis was conducted for 2 h using 5 V and 1 mA per 1 cm of strip width. To detect electrophoresis patterns, the cellulose acetate strips were stained with 0.1% Alcian blue (Sigma-Aldrich, Steinheim, Germany) solution in a mixture that was composed of 10 parts of 96% ethanol, 14 parts of water, and one part of glacial acetic acid for 30 min. Destaining was performed in a solution without stain. A quantitative analysis of the obtained electrophoretic patterns was conducted using the gel documentation system G:Box (Syngene, Cambridge, UK).

The circulating HA level was measured directly in the plasma samples using a pseudo-ELISA (enzyme-linked immunosorbent assay) (Chugai, Reads Medical Products, Westminster, CO, USA) that uses both native and enzyme-conjugated forms of the hyaluronan-binding protein to capture the GAG. The test was conducted according to the manufacturer’s protocol. The minimal detectable concentration of HA was 10 ng/mL. Because all of the plasma samples were measured in one day, the inter-assay variability was not estimated. In turn, the intra-assay variability of the HA concentration was less than 6%.

The data were analyzed using Statistica 12.0 Software (StatSoft Inc., Cracow, Poland). The normality of the distribution was verified by the Shapiro–Wilk test, whereas the variance homogeneity was analyzed by the Levene’s test. The data were summarized as the mean ± standard deviation (SD). Between-group comparisons were assessed based on a one-way ANOVA and the post hoc Tuckey’s test with *p* < 0.05 as significant. Moreover, a *t*-test was used to estimate the differences in the circulating HA levels between the patients with mild and severe AP. A Pearson correlation that used the *p*-values that were modified by the multi-testing Bonferroni correction was applied to evaluate the relationship between the circulating GAG levels and serum creatinine, bilirubin, and white cell count, as well as the total SOFA score in the mild and severe AP patients.

## 3. Results

The clinical–biochemical characteristics of all of the adult individuals who participated in the study are presented in Table 1.

It should be emphasized that the blood samples were drawn from the AP patients on the first day of their hospitalization. Moreover, because the number of AP patients with a moderately severe form of the disease (organ/system failure ≤48 h [18]) was small, their blood samples were not analyzed separately but were included in the group of those with severe AP.

**Figure 2:**
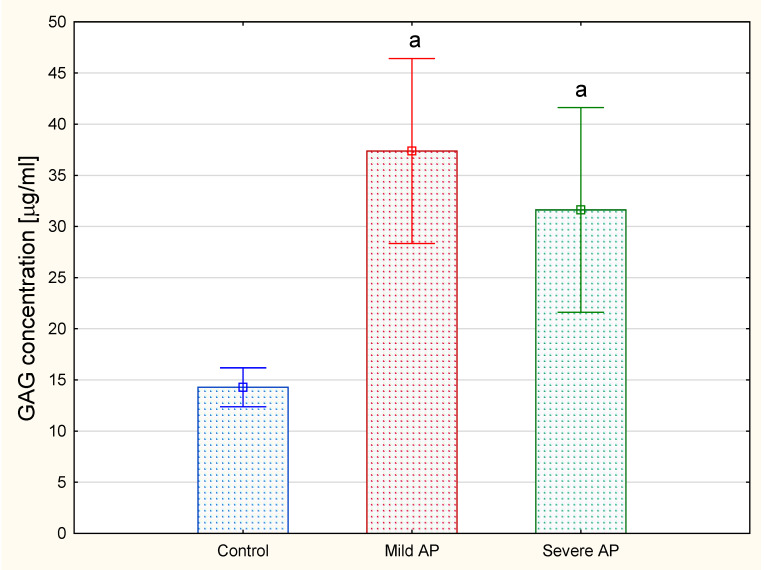
Total concentrations of glycosaminoglycans (GAGs) in the plasma of the healthy controls and the patients with mild or severe acute pancreatitis (AP). GAGs were isolated from plasma samples as described in Section 2 and were quantified by measuring the hexuronate concentration. Data are presented as means ± SD. a—differences statistically significant (*p* < 0.05) compared to the healthy controls.

The analysis revealed that the total plasma concentrations of the hexuronate-containing GAGs in both the patients with mild and severe AP were more than doubled compared to the levels of these macromolecules in the healthy controls (Figure 2). In order to identify the types of circulating GAGs, isolated molecules were subjected to electrophoresis on cellulose acetate before or after treatment with specific bacterial lyases.

**Figure 3:**
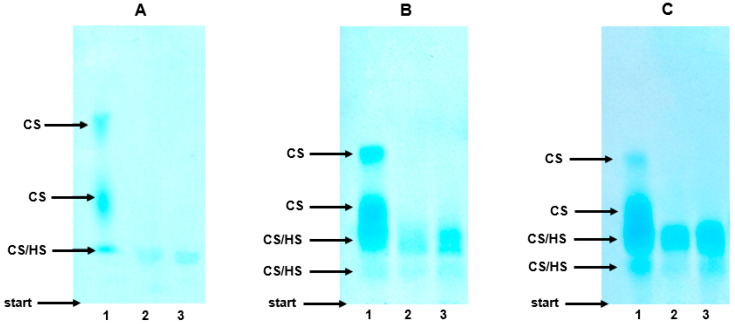
Typical electrophoretic pattern of the circulating glycosaminoglycans (GAGs) in the healthy controls (**A**), the patients with mild acute pancreatitis (**B**), and the patients with severe pancreatitis (**C**). GAGs were isolated from plasma samples as described in Section 2 and were submitted to electrophoresis on cellulose acetate in 0.017 M Al_2_(SO_4_)_3_ before and after treatment with bacterial lyases that specifically degrade some type of these macromolecules. Gels were stained with Alcian blue. Lane 1—untreated GAG sample; lane 2—GAG sample submitted to the combined action of chondroitinase ABC and heparinase I and III; lane 3—GAG sample treated with chondroitinase ABC.

As can be seen from Figure 3A, lane 1, the electrophoretic profile of the plasma GAGs in the healthy controls was composed of three molecular fractions that differed with respect to their migration rate. The species that migrated with the highest and intermediate electrophoretic mobility were identified as CS based on their complete sensitivity to the action of both chondroitinase ABC (Figure 3A, lane 3) and chondroitinase AC I, which was manifested by the disappearance of the two fastest bands in the treated samples (Figure 3A, lane 3 versus 1). In turn, chondroitinase ABC (or chondroitinase AC I) was able to eliminate only some of the compounds that migrated most slowly (Figure 3A, lane 3 versus 1). Moreover, the species that were resistant to the chondroitinase(s) showed only some sensitivity to the heparinases (Figure 3A, lane 3 versus 2). Thus, the slowest glycan fraction included both CS and HS together with a very small amount of material that was resistant to the used enzymes (Figure 3A, lane 2). To summarize, the electrophoretic analysis indicated that CS was a major GAG in the plasma of the healthy controls, whereas HS made only a small contribution into the plasma GAG profile (Figure 6). In addition, the plasma CS was characterized by a prominent structural heterogeneity most probably in respect to the sulfate content and/or chain size, which was reflected by the visible differentiation of the electrophoretic mobility of this GAG (Figure 3A, lane 1).

**Figure 4:**
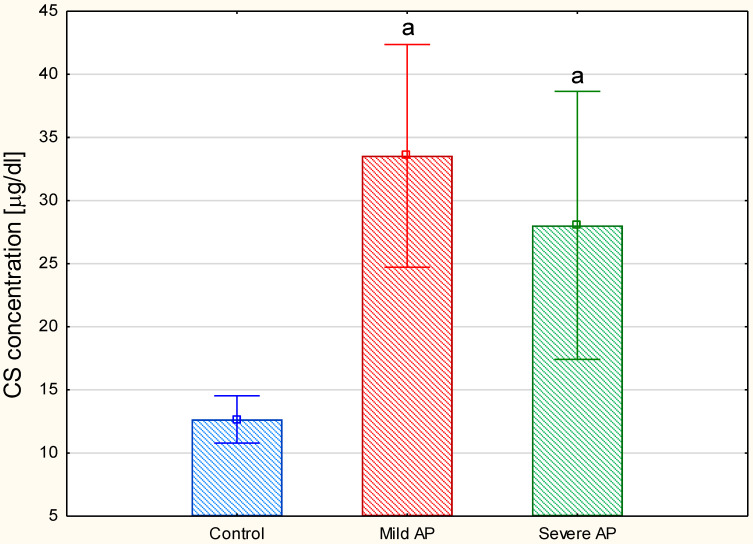
Concentrations of the circulating chondroitin sulfate (CS) in the healthy controls and the patients with mild or severe acute pancreatitis (AP). CS was identified in the samples of plasma glycosaminoglycans using a combination of electrophoresis on the cellulose acetate and specific degradation with chondroitinase ABC. Data are presented as means ± SD. a—differences statistically significant (*p* < 0.05) compared to the healthy controls.

**Figure 5:**
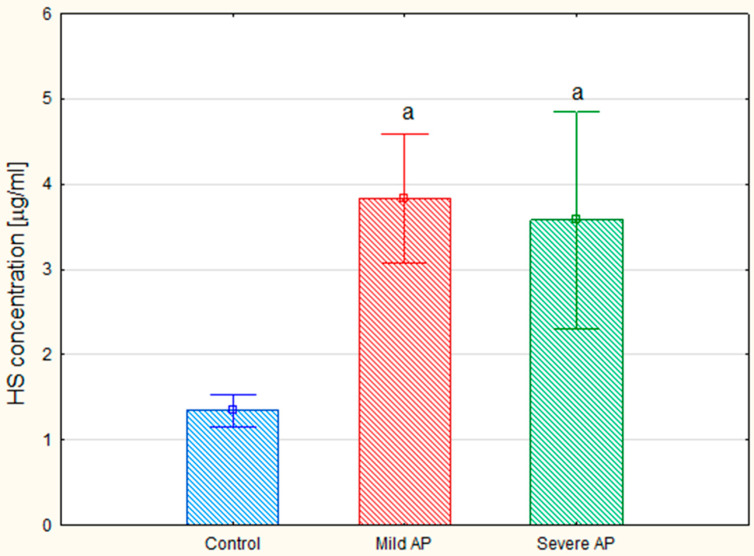
Concentrations of the circulating heparan sulfate (HS) in the healthy controls and the patients with mild or severe acute pancreatitis (AP). HS was identified in the samples of plasma glycosaminoglycans using a combination of electrophoresis on the cellulose acetate and treatment with heparinases I and III. Data are presented as means ± SD. a—differences statistically significant (*p* < 0.05) compared to the healthy controls.

**Figure 6:**
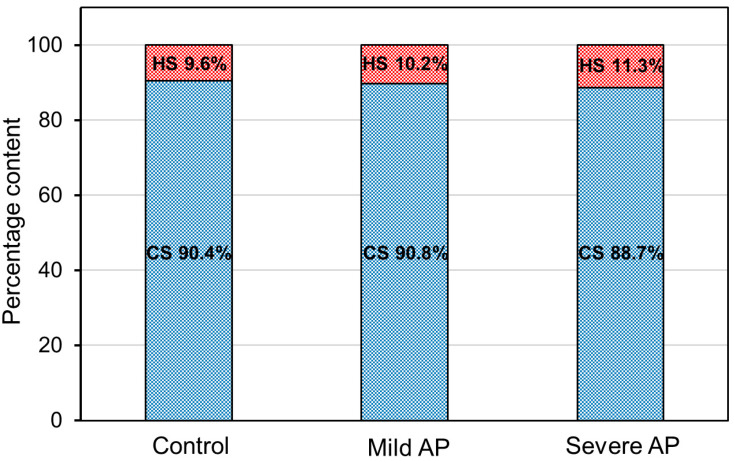
Comparison of the percentage proportions between the circulating chondroitin sulfate and circulating heparan sulfate in the healthy controls and the patients with mild or severe acute pancreatitis (AP).

The electrophoretic patterns of the circulating GAGs from both the patients with mild and severe AP also had three GAG-containing fractions and were rather similar to the electrophoretic profile that was found in the healthy controls (Figure 3B,C versus 3A, lanes 1). Only the species with an intermediate electrophoretic mobility migrated more diffusely in the AP patients compared to the control samples (Figure 3B,C versus 3A, lanes 1). However, like the controls, in the AP samples, CS was distributed into all three GAG fractions as resulted from their sensitivity to the action of chondroitinase ABC (Figure 3B,C, line 3 versus 1) or chondroitinase AC I. However, in contrast to the controls, in all of the AP samples, HS was not only found in the slowest glycan fraction, but was also among the species that migrated with an intermediate electrophoretic mobility, which was indicated by their partial sensitivity to heparinases (Figure 3B,C, lane 2 versus 3). Moreover, a significant increase in the amount of the material that was resistant to the used enzymes was also observed in all of the AP patients compared to the healthy donors (Figure 3B,C versus 3A, lanes 2). All of the data presented above suggest that the plasma GAG profile can be remodeled in all AP patients regardless of the form of the disease. This suggestion was further supported by the results of the quantitative analysis, which showed a statistically significant, more than two-fold increase in the circulating CS concentrations in all of the AP patients compared to the healthy controls (Figure 4). Interestingly, the mean CS level in the patients with severe AP was slightly lower than that in the patients with mild AP (28.04 mg/L versus 33.55 µg/mL, respectively); however, this difference was not statistically significant. Additionally, a nearly three-fold increase in the circulating HS levels was observed in all of the AP patients compared to the control samples (Figure 5). However, the proportions of circulating HS to circulating CS in the AP patients were similar to those that were found in the healthy controls (Figure 6).

**Figure 7:**
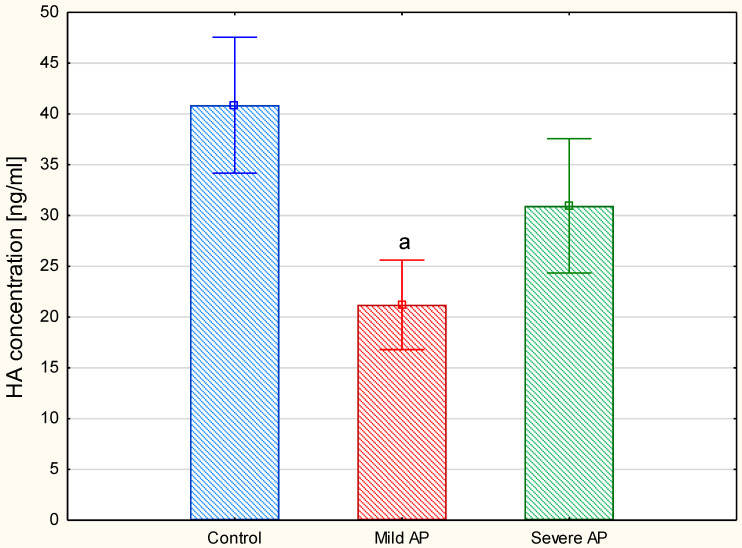
Concentrations of the circulating hyaluronan (HA) in the healthy controls and the patients with mild or severe acute pancreatitis (AP). HA was quantified in the plasma samples using ELISA-based test. Data are presented as means ± SD. a—differences statistically significant (*p* = 0.001) compared to the healthy controls.

Because the plasma HA concentration is usually below the detection threshold of the electrophoretic methods, we used an ELISA method-based test to measure this parameter. The comparison of the plasma HA levels between the three examined groups showed (Figure 7) that, in contrast to the patients with severe AP, those with a mild form of the disease had significantly decreased this parameter compared to the healthy controls. Moreover, the exclusion of the individuals with moderately severe AP (four patients) from the severe AP group did not significantly affect these between-group differences. However, a comparison of the circulating HA concentrations between only the two AP groups using a *t*-test clearly indicated that the patients with a mild disease also had a significantly lower (*p* = 0.012) level of this GAG in relation to the individuals with severe AP (i.e., the patients with moderately severe and severe AP together).

In order to evaluate the relationship between the remodeling of the plasma GAG profile and the actual clinical status of the patients with the different forms of AP, the correlations between the circulating GAG levels and the markers of renal (serum creatinine) and liver (serum bilirubin) function, as well as the SIRS (leucocyte count) and the total SOFA score, which were measured on the first day of the hospitalization, were analyzed. We selected those clinical parameters for several reasons. The functional status of the kidneys and liver helps to differentiate the mild and severe forms of AP and also plays a pivotal role in removing the circulating GAGs. In turn, SIRS is a crucial mechanism that is responsible for the induction and stimulation of organ dysfunctions in patients with severe AP. Moreover, the SOFA score is commonly used in clinical practice to evaluate the prognosis in critically ill patients and/or patients who are suffering from AP [17,18]. Thus, a potential relationship between the circulating GAGs and the examined markers and score should indicate the involvement of these molecules in the pathogenesis of AP and/or estimate their prognostic value for clinical practice. The obtained correlations are shown in Table 2. As the results from these data show, the circulating CS concentrations had a positive relationship with creatinine in the patients with the severe form of the disease (Table 2). Moreover, a positive correlation between their HA levels and the total SOFA score was also found in those patients (Table 2). By contrast, there was no significant correlation in the mild AP patients.

## 4. Discussion

Our data clearly show that a significant remodeling of the circulating GAG profile occurred not only in patients with severe AP but, surprisingly, also in patients with the mild form of the disease compared to the healthy controls, and that these changes can be detected early in the progression of AP. This finding suggests that systemic changes in the PG/GAG metabolism are not only elements of pathogenesis in a severe AP but also in the mild disease. Interestingly, the quantitative changes in the circulating GAGs that were found in the mild and severe AP patients had nearly similar manifestations. They were reflected in (1) a more than two-fold increase in the total plasma concentration of the hexuronate-containing GAGs, (2) a significant augmentation of the circulating CS concentration, and (3) a marked increase in the circulating HS level and an increase in the structural diversity of this GAG, which affected its electrophoretic mobility. Only the levels of plasma HA were different in the patients with mild and severe AP. The observed remodeling of the circulating GAGs had a distinct feature apart from several similarities when it was compared to the changes in GAG profiles that were recorded in the plasma of patients suffering from acute SIRS-associated disorders such as septic shock or severe respiratory failure due to sepsis or AP [15,16,21]. This difference was related to the circulating CS concentration. In contrast to our results, the plasma levels of CS that were observed in those patients were similar to the concentrations of this GAG in the healthy controls [16,21]. Importantly, severe AP and sepsis share the basic elements of their pathogenesis such as the activation of innate immunity, SIRS, and multi-organ dysfunctions [22]. However, our AP patients and those suffering from septic shock or severe respiratory failure due to sepsis or AP [16,21] differed in respect to both the duration of the disease and its severity (for example, patients with respiratory distress were mechanically ventilated [21]). Moreover, we also employed different methods for isolating and detecting the circulating CS than the above-mentioned investigators. On the other hand, our patients with severe AP also displayed a trend to decrease the circulating CS levels compared to the patients with a mild form of the disease. Additionally, the CS-containing plasma species in our AP patients with mild and severe AP may differ in respect to some biological properties. This conclusion results from our observation that only in the patients with severe AP the circulating CS was positively correlated with serum creatinine, thus suggesting that this GAG-containing molecules might be removed via renal clearance.

Growing evidence indicates that the CS-containing plasma species can play a crucial role in the inflammation-associated diseases. It is well known that an essential if not a main part of plasma CS is formed by members of the inter-α-inhibitor family, i.e., pre-α-inhibitor (PαI) and inter-α-inhibitor (IαI), which are synthesized in the liver and both of which are precursors of another CS-containing plasma molecule called bikunin [23,24]. Interestingly, PαI and IαI have strong anti-inflammatory effects, which manifest as inhibiting of neutrophil activation [25], protecting against histone-induced coagulopathy and organ damage [26], as well as attenuating the complement activation and complement-dependent lung injury [27]. In turn, bikunin, which is liberated from IαI and PαI as a result of the transfer of the so-called heavy chain(s) mainly to HA, displays an inhibitory activity toward several serine proteases such as plasmin, trypsin, cathepsine G, or human leucocyte elastase [23,24], thereby having a weak anti-inflammatory effect. Moreover, in contrast to other members of the IαI family, owing to its low molecular mass, free bikunin is quickly eliminated from the circulation via glomerular filtration [24]. The metabolism of IαI glycoproteins undergoes marked changes in inflammation-associated illnesses, which results from the altered expression profiles of these molecules in the liver (i.e., a strongly stimulated expression of PαI and a downregulation of the bikunin precursor and IαI [28]) and their increased consumption under inflammatory conditions. Thus, the dynamic balance between the synthesis of the CS-containing IαI glycoproteins and their use, which also depends on the liver and renal functions, can significantly modulate the course of inflammatory diseases.

In addition to the alterations in the metabolism of the IαI glycoproteins, another factor that can influence the pool of the circulating CS under inflammatory conditions is the destruction of the glycocalyx of the endothelial cells [29]. However, the biological consequences that result from the appearance of degradation products of the cell surface chondroitin sulfate proteoglycans in the circulation remain unclear.

Similar to the plasma concentrations of CS, the levels of circulating HS were markedly higher not only in our patients with severe AP but, surprisingly, also in those with a mild form of the disease compared to the healthy controls. On the other hand, the data that concern the severe AP patients are in line with the findings of others who showed that an increased HS concentration is a sign of a remodeling of the circulating GAG profile in acute and chronic severe SIRS-associated illnesses [12,16,21]. It should be emphasized that a similar character of this remodeling was even observed despite the differences in the methods for isolating and/or detecting HS that were used by us and some of the above-mentioned investigators [16,21]. The mechanism that underlies this increase in the circulating HS level might involve a TNF-α-induced, systemic upregulation of heparanase in the vascular endothelial cells [30]. This enzyme, whose activity was found to be increased in the blood of patients with sepsis or severe AP [21], participates in the degradation of the glycocalyx of blood and endothelial cells, which results in the release of HS [11]. Interestingly, a local pancreatic increase of both the heparanase expression and activity was also observed in a mouse model of caerulein-induced mild AP [30]. Thus, the degradation of pancreatic heparan sulfate proteoglycans (HSPGs) may be responsible for delivering the HS-containing species to the circulation in patients with mild AP (and, obviously, in individuals with severe AP). However, it is unlikely that this inefficient process could induce such great changes in the level of the circulating HS as those that were found in our patients with mild AP. Thus, additional investigations are needed to elucidate the possible source(s) of the circulating HS in mild AP.

It is believed that plasma HS may be involved in aggravating SIRS-associated illnesses, since HSPG degradation products are considered to be damage-associated molecular pattern ligands, which induce inflammatory signaling and cytokine upregulation via interactions with Toll-like receptor 4 (TLR4) and/or TLR2 [11]. In addition, fragments of the tissue HS chains can affect the biological activity of many growth factors and cytokines by modulating their interactions with the appropriate cell surface receptors [9,11]. The crucial role of HS in both the induction and exacerbation of AP was further evidenced in in vivo investigations using animal models [31,32]. However, neither we nor others who examined the concentration of plasma HS in patients with septic shock or with respiratory failure due to sepsis or severe AP [16,21] found any significant correlation between this parameter and several clinical markers such as the creatinine and bilirubin concentrations or leucocyte counts. Only the total SOFA score was found to positively correlate with the circulating HS concentrations in patients with septic shock [16]. Summarizing our data referring to the plasma HS, we conclude that factors that regulate the level and biological properties of this GAG are extremely complex not only in systemic inflammation-associated severe AP, but also in a mild form of the disease that is connected with a local inflammatory process.

Our results clearly show that changes in the plasma levels of sulfated GAGs (i.e., CS and HS) have no relevance in stratifying the AP severity. However, our results also indicate for the first time that the circulating HA level can be a promising parameter that enables mild AP to be distinguished from severe disease early, which can be done by simply comparing the results that are obtained for an AP patient to the reference interval for this GAG. This conclusion can be drawn from the observation that only the patients with mild AP demonstrated a significantly lower plasma concentration of this GAG compared to the healthy controls, whereas the patients with a severe disease had the normal level of circulating HA. The last finding is also in line with the observation of Schmidt et al. [21], who examined this marker in individuals with respiratory failure due to sepsis or severe AP. However, it should be kept in mind that these investigators used a different method to detect HA than ours. In turn, patients with severe sepsis or septic shock had significantly higher plasma concentrations of HA or HA-derived disaccharides than the non-septic critically ill individuals or healthy controls, respectively [16,33]. Summing up, all of these findings suggest that the HA metabolism is strongly associated with the intensity and extent of inflammation. It is well evidenced that HA deposition is significantly increased in inflamed tissues including the vascular wall, and that this process is accompanied by an increased modification of the GAG via its coupling to heavy chains (HC) [34,35] of which the primary donors are the IαI glycoproteins [23,24,34]. This modification of the HA network induces the recruitment of leukocytes and their adhesion to this GAG, thereby promoting inflammation [36]. On the other hand, when HCs are linked to high-molecular-mass HA, they protect this GAG against degradation [23,24]. This latter process is the source of the products that have strong pro-inflammatory effects, which are triggered via the activation of the TLR4 and the hyaluronan receptor for endocytosis, as well as the upregulation of the inflammatory cytokines [37,38]. Moreover, the increased degradation of high-molecular-weight HA leads to an increase in the circulating fraction of this GAG, which is also controlled via a liver-dependent clearance [39]. Thus, regulating the stability of high-molecular-weight HA may be an important mechanism that controls the progression of an inflammatory disease. On the other hand, the involvement of the HA degradation products in the amplification of inflammation, which can lead to SIRS-induced (multi)organ failure, might be the reason for the strong positive correlation between the plasma HA concentration and the total SOFA score that we observed in the patients with severe AP. Interestingly, a similar relationship between these parameters was also found in other critically ill patients [33]. However, a strong positive association between the plasma HA level and total SOFA might partly result from the involvement of some SOFA components in the metabolism of the GAG. On the other hand, the lack of a significant relationship between the circulating HA level and the liver function in our AP patients might be due to the fact that the functional status of this organ was assessed only by serum bilirubin concentration, which is a part of the SOFA score. However, serum bilirubin level does not adequately reflect the actual liver function in patients with gallstone-induced AP. Individuals with this etiology constituted half of our AP patients. Therefore, the estimation of the association between the plasma HA concentration (or the plasma levels of the remaining GAGs) and the liver function in AP patients should be revised in future studies by using additional parameters that describe this organ activity.

## 5. Conclusions

Our data suggest that, apart from its value as an early prognostic marker of AP evolution, the circulating HA level can be used to evaluate (or to monitor) the clinical status of patients with the severe form of the disease. However, the clinical value of the circulating HA level, especially to predict the severity of AP, requires a verification that not only takes into account the individual influence of various etiological factors and the degrees of the severity of the disease on this parameter but that will also be based on an analysis of many samples using matched controls. Moreover, the influence of a delayed or atypical presentation of AP on the circulating HA should also be tested.

## Figures and Tables

**Table 1:** Laboratory/clinical characteristic of healthy controls and patients with acute pancreatitis (AP).

Parameter	Healthy Controls	Patients with A Mild AP	Patients with A Severe AP
Number of individuals (*n*)	23	32	15 (moderately severe—4; severe—11)
Age (years)	50 * (23–85)	48 * (28–85)	53 * (22–87)
Gender male/female	15/8	22/10	10/5
AP etiology—alcohol abuse/cholelithiasis	-	17/15	8/7
Serum amylase (IU/L)	93 * (20–144)	1689 * (514–8625)	2327 * (503–10,158)
Serum lipase (IU/L)	36 * (18–57)	509 * (206–2126)	1562 * (493–8993)
White blood cells (10^9^/L)	7.90 ^•^ ± 3.12	13.03 ^•^ ± 3.91	15.15 ^•^ ± 4.82
Serum creatinine (mg/dL)	0.84 ^•^ ± 0.23	1.10 ^•^ ± 0.23	1.51 ^•^ ± 0.81
Total serum bilirubin (mg/dL)	0.6 ^•^ ± 0.28	1.12 ^•^ ± 0.59	2.07 ^•^ ± 1.52
Total SOFA score	-	0 * (0–2)	5 * (2–11)
Organ/system failure	-	-	Respiratory (*n* = 6) Liver (*n* = 3) Renal (*n* = 2) Multi-organ (*n* = 4)
Local complications	-	-	Pancreatic necrosis (*n* = 4) Pancreatic pseudocyst (*n* = 1)
Death	-	-	*n* = 4
Treatment in the ICU	-	-	*n* = 10
Length of hospitalization (days)	-	8 * (7–10)	21 * (5–29)

^•^ Results are expressed as means ±SD; * results are expressed as medians (range); SOFA—Sequential Organ Failure Assessment; ICU—intensive care unit.

**Table 2:** Pearson’s coefficients that describe the relationship between the plasma concentrations of glycosaminoglycans and the laboratory/clinical parameters in the patients with mild and severe acute pancreatitis (AP) at admission day.

Parameters	The Glycosaminoglycan Concentrations in the Plasma of the Patients with an AP					
	CS (μg/mL)		HS (μg/mL)		HA (ng/mL)	
	Mild AP	Severe AP	Mild AP	Severe AP	Mild AP	Severe AP
White cell count	−0.510 ^NS^	−0.053 ^NS^	0.116 ^NS^	−0.570 ^NS^	−0.227 ^NS^	−0.126 ^NS^
Creatinine	−0.479 ^NS^	0.879 (*p* = 0.016)	0.071 ^NS^	−0.641 ^NS^	−0.064 ^NS^	0.469 ^NS^
Bilirubin	0.039 ^NS^	0.335 ^NS^	0.364 ^NS^	−0.035 ^NS^	0.532 ^NS^	0.130 ^NS^
Total SOFA score	−0.096 ^NS*^	0.628 ^NS^	0.361 ^NS*^	0.477 ^NS^	0.288 ^NS*^	0.745 (*p* = 0.008)

AP—acute pancreatitis; CS—chondroitin sulfate; HS—heparan sulfate; HA—hyaluronan; SOFA—Sequential Organ Failure Assessment; NS—non-significant; * only four patients had a total SOFA score higher than 0 (two individuals with a score of one and two individuals with a score of one + one).
